# Supplementary material for: Development and characterization of functional antibodies targeting NMDA receptors
Source: Nat Commun. 2022 Feb 17;13:923. doi: 10.1038/s41467-022-28559-3 (PMC8854693; doi:10.1038/s41467-022-28559-3)
Supplement: Supplementary file 1 — Supplementary Information [file 41467_2022_28559_MOESM1_ESM.pdf]

Supplementary Information

Title: Development and characterization of functional antibodies targeting NMDA receptors

Authors:

Nami Tajima[1*], Noriko Simorowski[1*], Remy A. Yovanno[2*], Michael C. Regan[1], Kevin Michalski[1], Ricardo Gómez[1], Albert Y. Lau[2✓], and Hiro Furukawa[✓1]

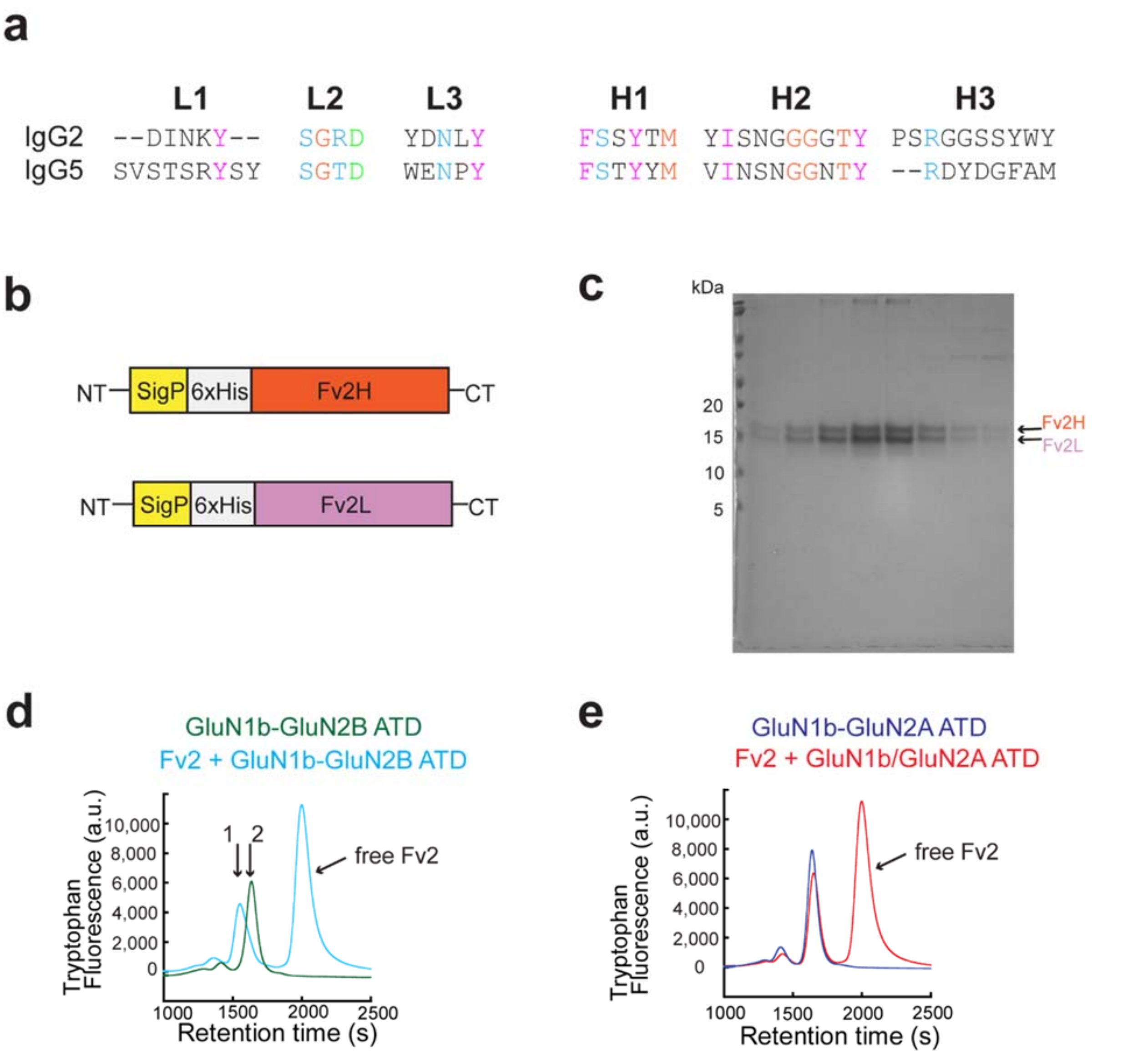

Supplementary Fig. 1. Preparation of Fv2 proteins

a Primary sequences of complementary determining regions (CDRs) from the heavy chain (H1-3) and the light chain (L1-3). **b** The Fv regions of IgG2 (Fv2) from the heavy (Fv2H) and light (Fv2L) chains are subcloned into the pNC-HisT vector (Takara) and recombinantly expressed in *Brevibacillus choshinensis.* The construct contains the modified signal peptide from HWP cell wall protein (SigP) followed by a hexa-histidine tag (6xHis) and the Fv2H or Fv2L. **c** The peak fractions of the Superdex200 size exclusion chromatography column. The molecular weights of Fv2H and Fv2L are predicted to be 16.0 kDa and 14.6 kDa, respectively, based on amino acid compositions and are consistent with the band pattern in the 20% SDS-PAGE. **d-e** The purified Fv2 proteins are capable of specifically interacting with the GluN1-GluN2B ATD proteins but not with the GluN1-GluN2A ATD proteins as monitored by FSEC using Superdex200 and intrinsic tryptophan fluorescence as the detection method (280/330 nm = excitation/emission).

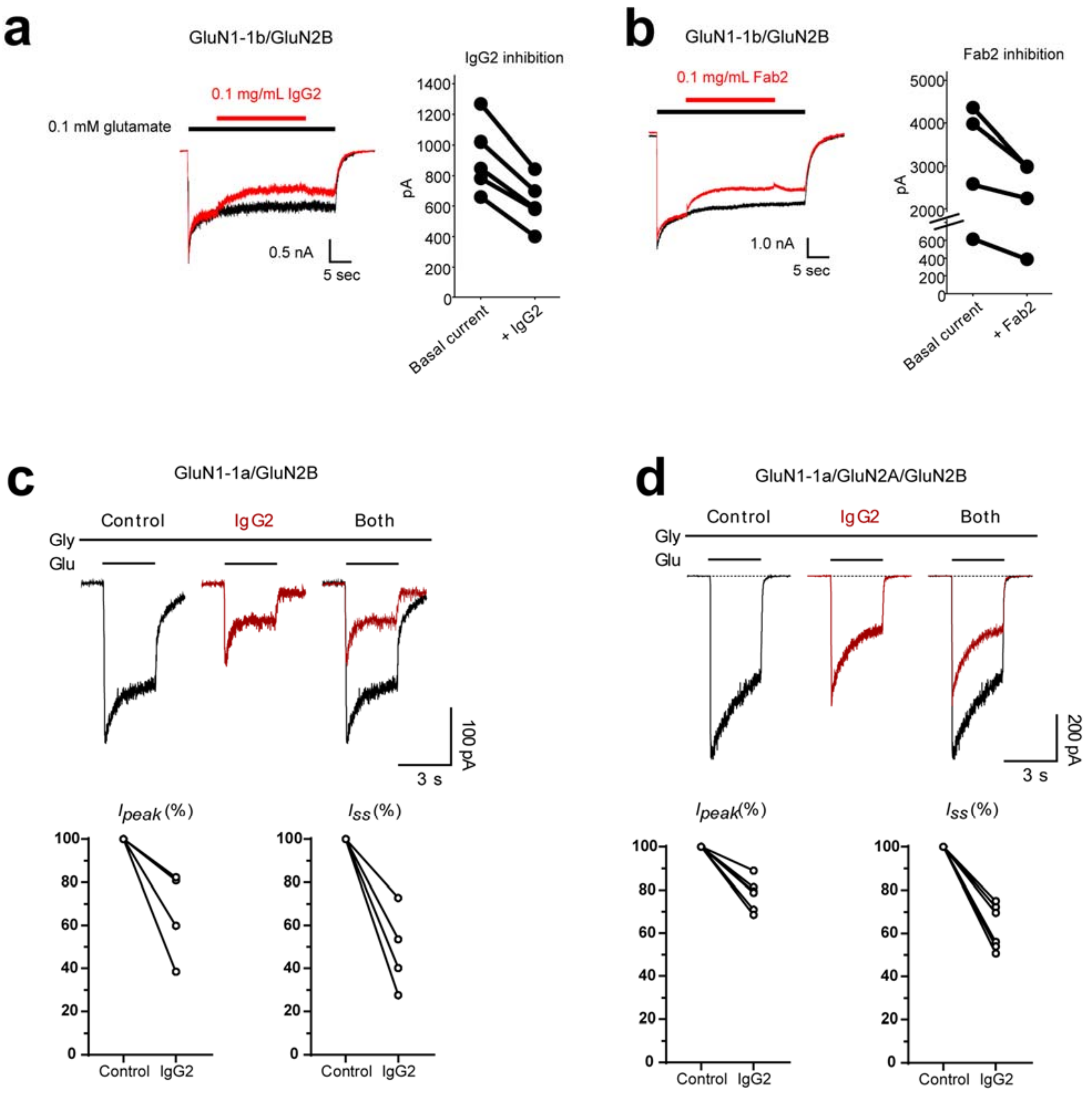

Supplementary Fig. 2. Inhibition of GluN1-GluN2B NMDAR by IgG2 and Fab2 in HEK293T cells a-b Whole-cell patch-clamp recordings of HEK293T cells transfected with GluN1b-GluN2B NMDAR. Cells were held at -80 mV under the tonic presence of 100 µM glycine and exposed to a solution containing 100 µM glutamate (black) using a rapid solution exchanger. In a second recording (red), cells were exposed to an additional solution containing 100 µM glutamate and 0.1 mg/ml IgG2 (**a**) or Fab2 (**b**), and the two traces were superimposed (n=5 and 4 patches, for IgG2 and Fab2, respectively). **c-d** The effect IgG2 on GluN1a-GluN2B NMDAR (n=4) (**c**) and GluN1a-GluN2A-GluN2B NMDAR (n=6) (**d**) before and after incubation with 0.1 mg/ml IgG2. For GluN1a-GluN2B NMDAR, peak current (I_{peak}) = 52.1 ± 19.2 pA/pF vs 28.6 ± 8.8 pA/pF (IgG2), and steady-state current (I_{ss}) = 23.6 ± 8.4 pA/pF (control) vs 8.8 ± 2.7 pA/pF (IgG2). For GluN1a-GluN2A-GluN2B NMDAR, I_{peak} = 88.9 ± 6.4 pA/pF (control) vs 69.6 ± 5.9 pA/pF (IgG2) and I_{ss} = 46.7 ± 5.1 pA/pF (control) vs 28.9 ± 2.8 pA/pF (IgG2). The values are mean ± SE.

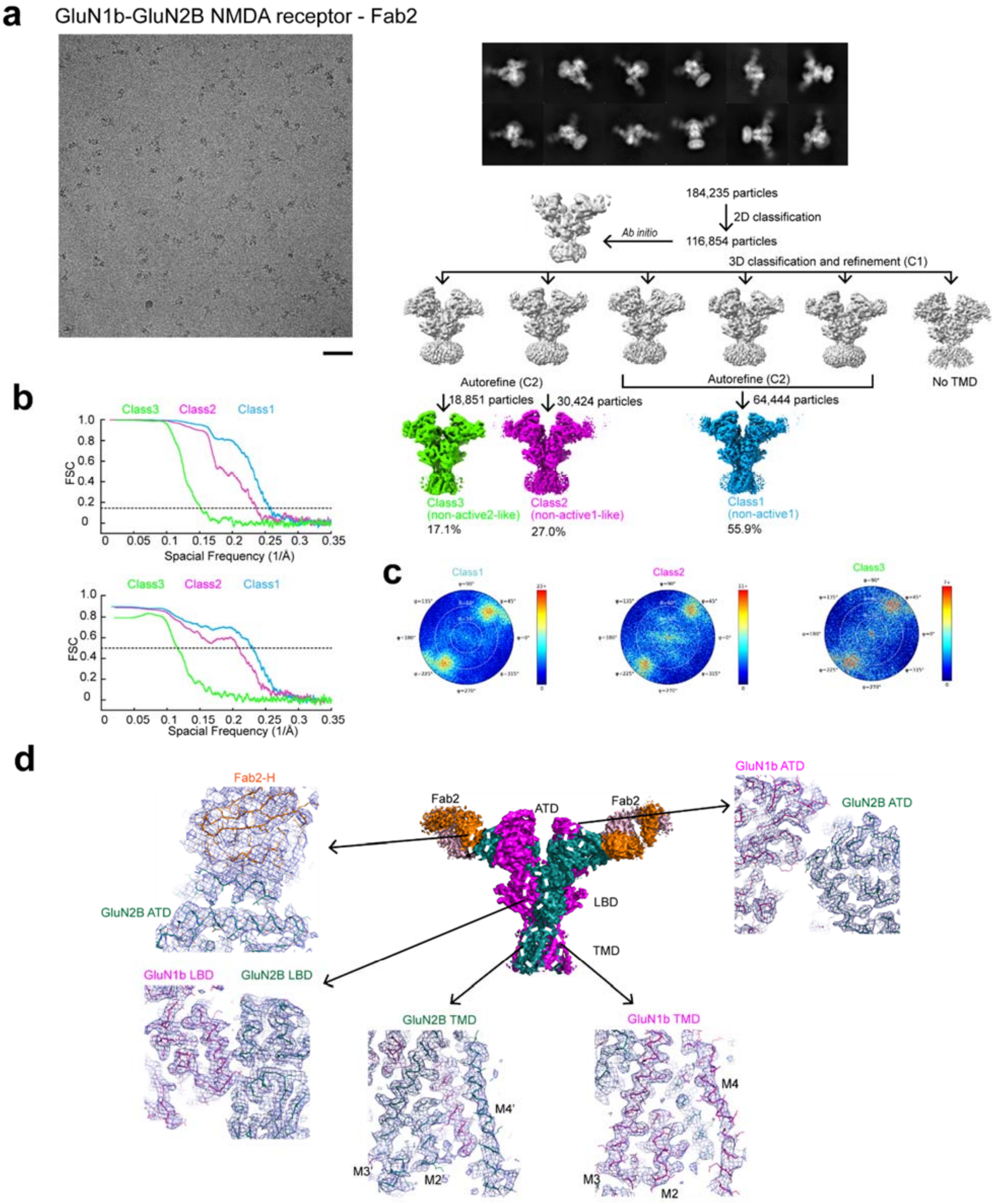

Supplementary Fig. 3. Single particle analysis on the GluN1b-GluN2B NMDAR-Fab2 complex. a A representative image, 2D classes, and the 3D classification workflow. The three major classes, Class1, 2, and 3 are closely related to non-active1, non-active1-like, and non-

active2-like, respectively. **b** FSC curves of two half maps (top) and map vs model (bottom). **c** The angular distribution plots for each class. **d** Zoomed-in views of cryo-EM density in each domain and subunit.

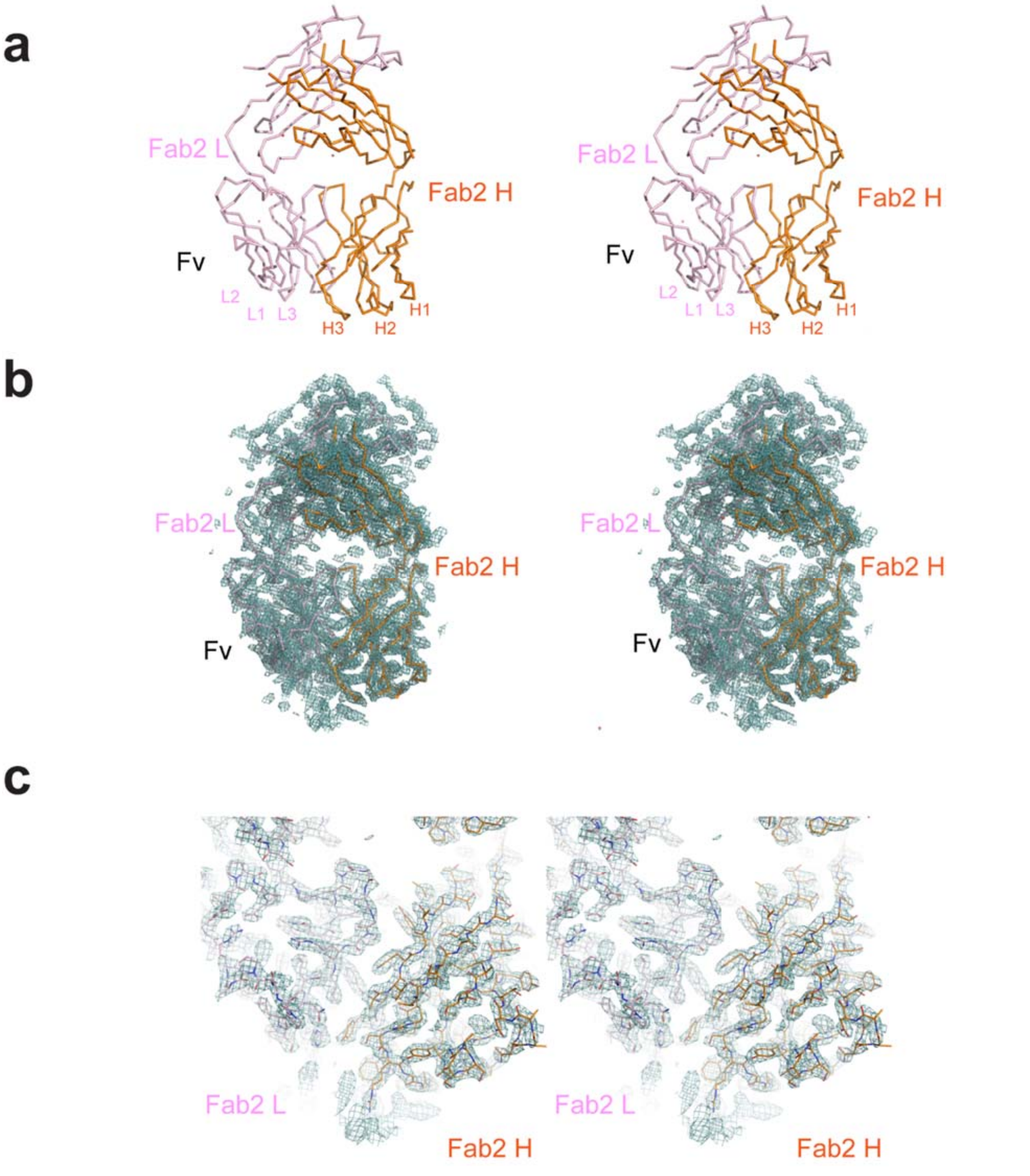

Supplementary Fig. 4. Crystal structure of Fab2 at 2.5 Å. a Stereoviews of the entire Fab2 fragment. The heavy and light chains are colored in orange and light pink, respectively. **b-c** Electron density of the entire Fab2 fragment (b) and the Fv region (c) showing sufficient quality to model CDR residues.

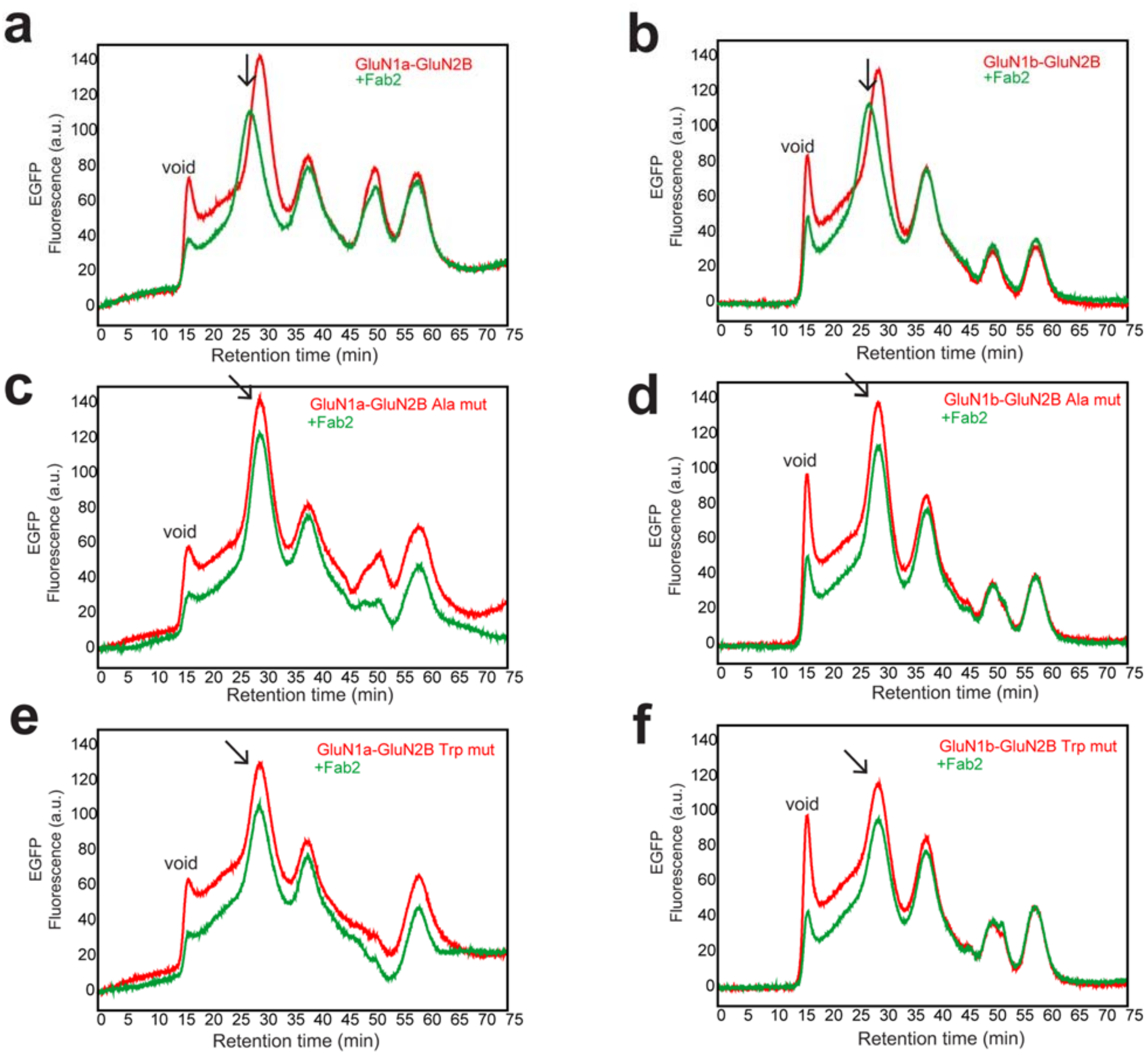

Supplementary Fig. 5. Effects of mutants on binding of Fab2 to GluN1-GluN2B NMDAR. The wild type (**a-b**), triple alanine mutant GluN2B Asp58Ala/His60Ala/Arg67Ala (Ala mut; **c-d**) and triple tryptophan mutant GluN2B Asp58Trp/His60Trp/Arg67Trp (Trp mut; **e-f**) were tested I the presence (green) and absent (red) of Fab2 (0.1 mg/ml). GluN1a-EGFP (a, c, and e) or GluN1b-EGFP (b, d, and f) into HEK293 cells. The solubilized samples were subjected to FSEC using EGFP fluorescence. The arrows indicate the tetrameric peak. Peak shift by Fab2 was observed only in the wildtype.

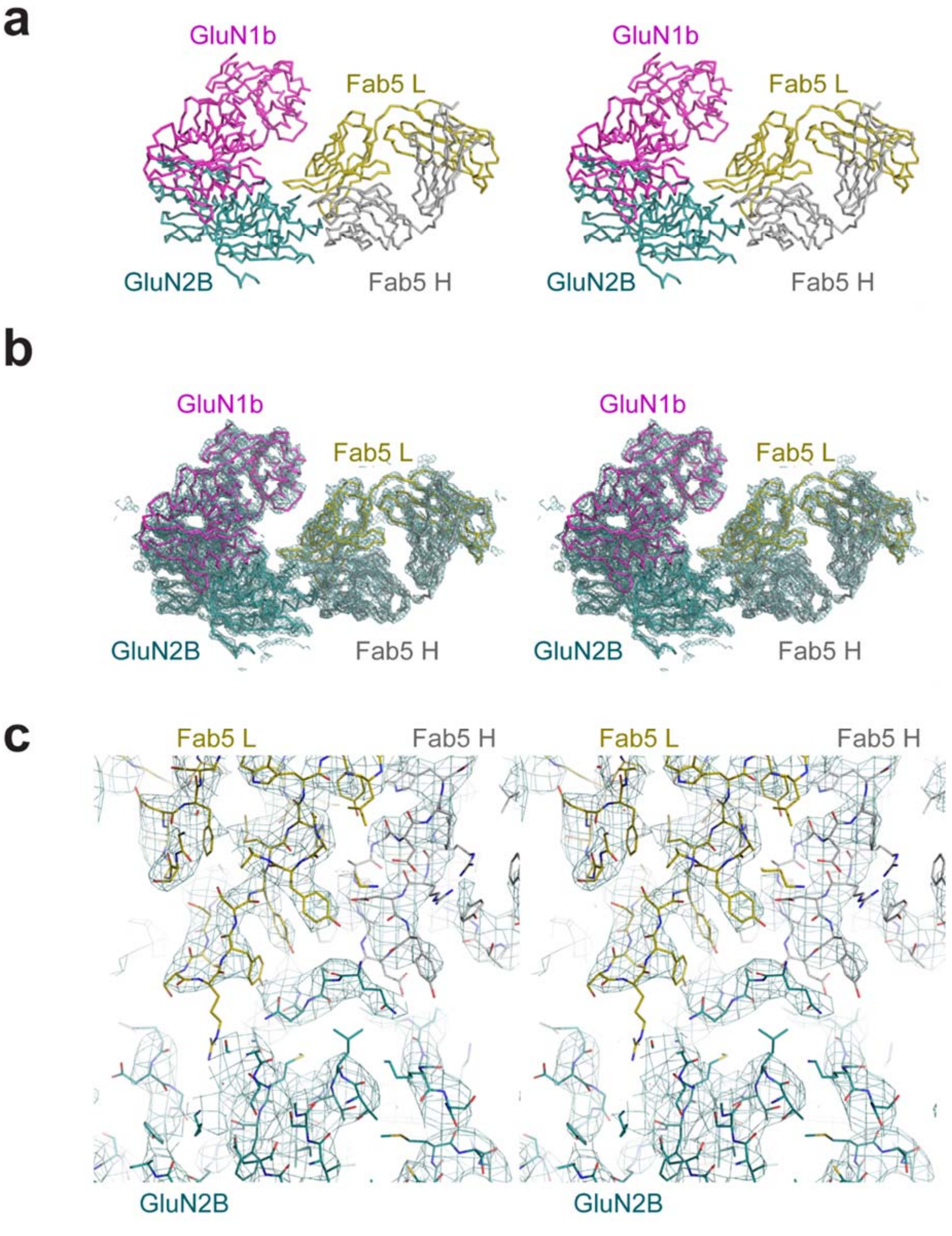

Supplementary Fig. 6. Crystal structure of GluN1b-GluN2B ATD-Fab5 complex at 4.55 Å. a Stereoviews of the entire GluN1b-GluN2B ATD-Fab5 complex. GluN1b, GluN2B, Fab5 H (heavy chain), and Fab5 L (light chain) are colored in magenta, dark green, gray, and dark gold, respectively. **b-c** Electron density of the entire complex (b) and the interaction site between GluN2B and Fab5 (c) showing mostly sufficient quality to model interacting residues.

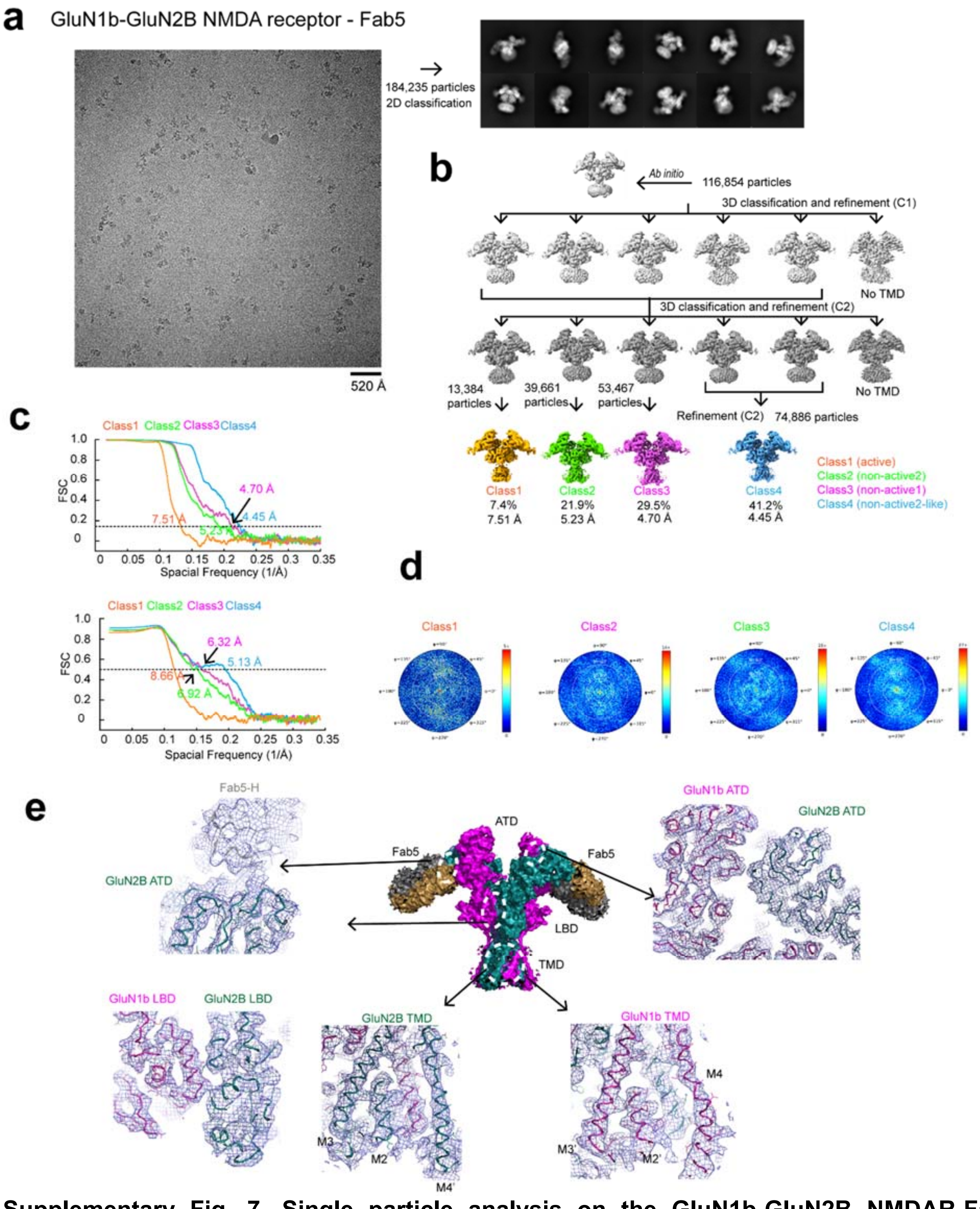

Supplementary Fig. 7. Single particle analysis on the GluN1b-GluN2B NMDAR-Fab5 complex. a-b A representative image, 2D classes, and the 3D classification workflow. The three major classes, Class1, 2, and 3 are closely related to non-active1, non-active1-like, and non-active2-like, respectively. **c** FSC curves of two half maps (top) and map vs model (bottom). **d** The angular distribution plots for each class. **e** Zoomed-in views of cryo-EM density in each domain and subunit.

a

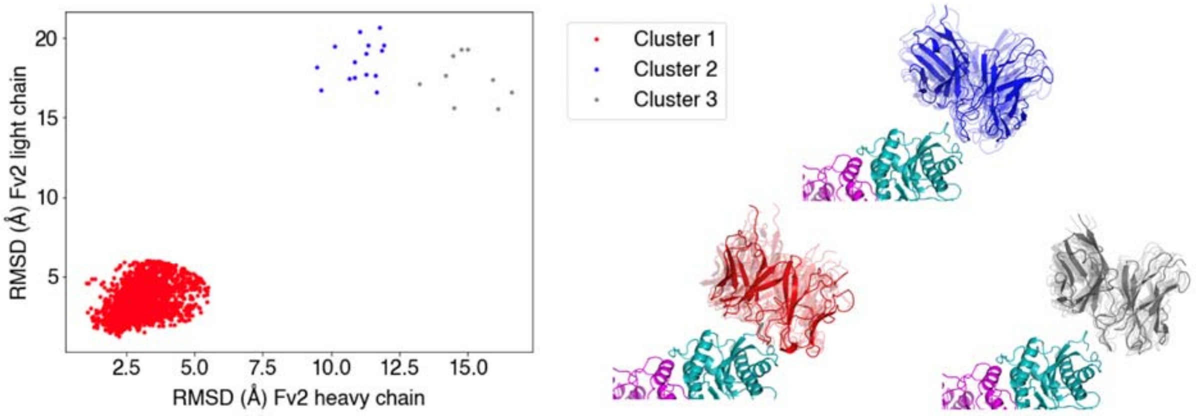

b

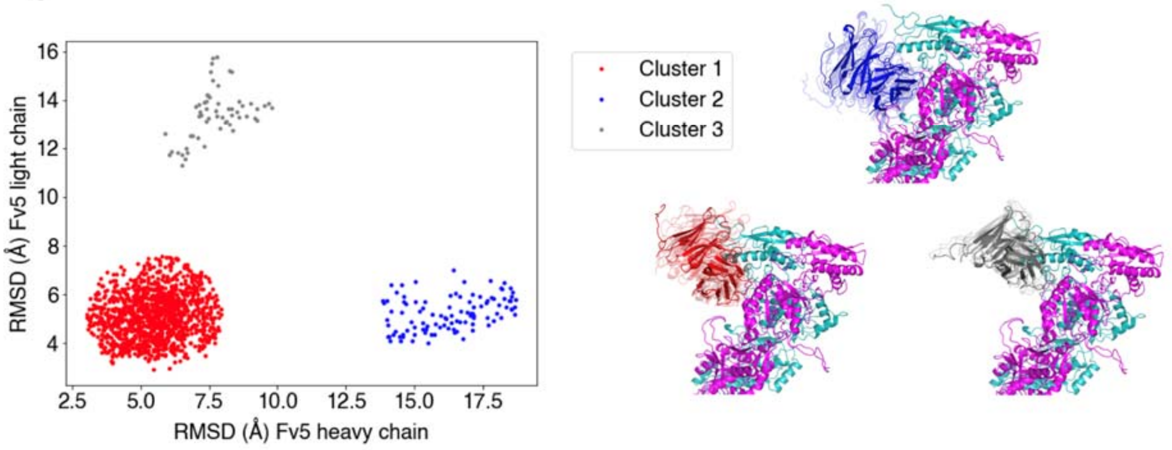

Supplementary Fig. 8. Stability of Fv binding to ATD. a-b Distinct binding modes of Fv2 (**a**) and Fv5 (**b**) determined from mean shift clustering. Simulation trajectories were clustered according to a two-dimensional feature set corresponding to the Cα RMSD of the heavy and light chain CDR loops. GluN1b ATD and GluN2B ATD are in magenta and green, respectively. Fv2 and 5 in Cluster1, 2, and 3 are in red, blue, and gray.

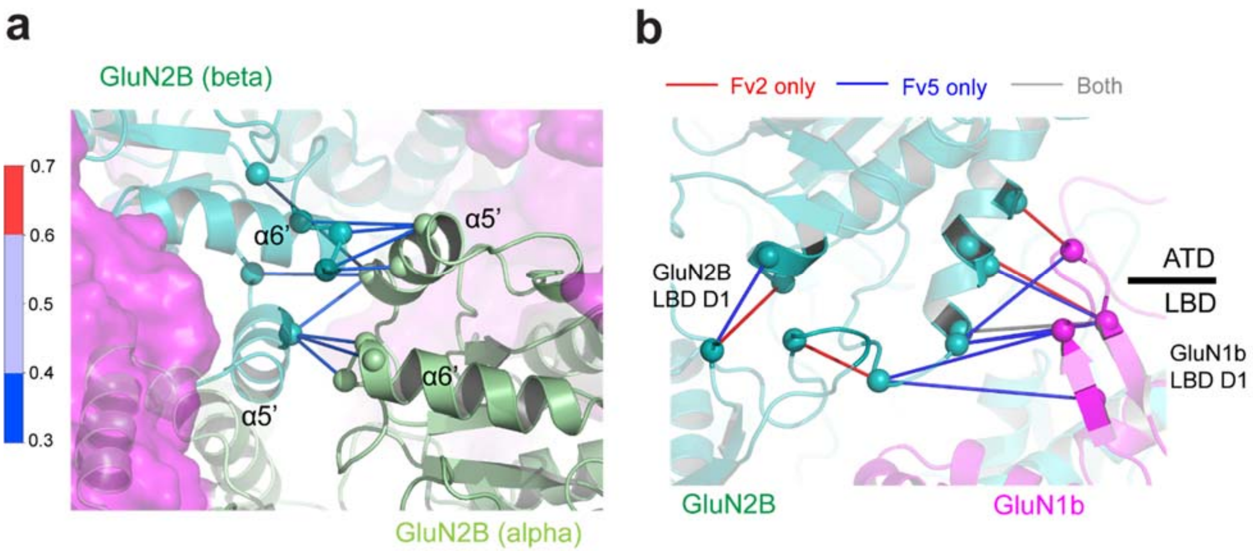

Supplementary Fig. 9. Other NMDAR interface contacts stabilized by Fv binding. a The inter-subunit interactions between the two GluN2Bs (alpha and beta subunits colored in lime and green, respectively) in the ATD layer of the hetero-tetramer, which are significant in simulations of the Fv5-bound NMDAR but not Fv2-bound NMDAR. Key interactions and secondary structure elements are labeled, and edge correlations are shown. Since the interactions are present in fewer simulation windows, we show the average correlation coefficients of nonzero windows and expanded our criteria to include contacts that meet the network criteria for at least 10/20 simulation windows. **b** Inter-domain interactions between the GluN2B ATD R2 lobe and both GluN1b and GluN2B LBDs (D1 lobes) labeled by their presence in simulations of Fv2 (red), Fv5 (blue), or both (gray). For Fv2, values are computed from simulations of an alternate conformation of the ATD-LBD linker for interactions present in >=7/10 of simulation windows.

Supplementary Table 1. Cryo-EM data collection, refinement and validation statistics for GluN1b-GluN2B NMDA receptors – Fab2

	Non-active1 (EMDB-25843) (PDB 7TE9)	Non-active1-like (EMDB-25844) (PDB 7TEB)	Non-active2-like (EMDB-25845) (PDB 7TEE)
**Data collection and processing**			
Magnification	105,000	105,000	105,000
Voltage (kV)	300	300	300
Electron exposure (e–/Å^2)	65	65	65
Defocus range (μm)	1.5 – 3	1.5 – 3	1.5 – 3
Pixel size (Å)	1.37	1.37	1.37
Symmetry imposed	C2	C2	C2
Initial particle images (no.)	184,235	184,235	184,235
Final particle images (no.)	64,444	30,424	18,851
Map resolution (Å)	3.92	4.23	6.59
FSC threshold	0.143	0.143	0.143
Map resolution range (Å)			
**Refinement**			
Initial model used (PDB code)	6CNA Crystal structure of Fab2	6CNA Crystal structure of Fab2	6CNA Crystal structure of Fab2
Model resolution (Å)	4.34	4.84	7.93
FSC threshold	0.5	0.5	0.5
Model resolution range (Å)			
Map sharpening *B* factor (Å^2)	-90	-90	-90
Model composition			
Non-hydrogen atoms	28,142	28,161	28,054
Protein residues	3,601	3,580	3,567
Ligands	0	0	0
CC map vs. model (%)	74	69	75
R.m.s. deviations			
Bond lengths (Å)	0.006	0.007	0.006
Bond angles (°)	1.061	1.457	1.422
Validation			
MolProbity score	2.09	2.20	2.69
Clashscore	8.61	7.85	9.80
Poor rotamers (%)	1.21	1.52	5.61
Ramachandran plot			
Favored (%)	89.59	86.54	87.43
Allowed (%)	9.79	12.22	11.55
Disallowed (%)	0.62	1.25	1.03

Supplementary Table 2. Data collection and refinement statistics

	Fab2 (PDB 7TE4)	GluN1b-GluN2B ATD – Fab5 (PDB 7TE6)
**Data collection**		
Space group	$P2_12_12_1$	$P4_12_12$
Cell dimensions		
a, *b*, *c* (Å)	54.43, 66.87, 240.25	124.92, 124.92, 407.12
α, β, γ (°)	90.00, 90.00, 90.00	90.00, 90.00, 90.00
Resolution (Å)	2.46 (2.499) *	4.54 (4.62)*
R_{sym} or R_{merge}	0.182 (1.168)	0.156 (1.107)
$I / \sigma I$	10.3 (2.1)	6.2 (1.1)
Completeness (%)	99.9 (100)	97.5 (86.5)
Redundancy	7.8 (7.6)	7.7 (3.4)
**Refinement**		
Resolution (Å)	30.0 – 2.50	25.0 – 4.55
No. reflections	33,153	17,965
R_{work} / R_{free}	26.1 / 29.6	28.2 / 32.4
No. atoms		
Protein	6,017	17,193
Ligand/ion		
Water	6	-
B-factors		
Protein	46.4	83.8
Ligand/ion		
Water	41.5	-
R.m.s. deviations		
Bond lengths (Å)	0.008	0.007
Bond angles (°)	1.083	1.137

*Each dataset was collected from a single crystal. *Values in parentheses are for highest-resolution shell.

Supplementary Table 3. Cryo-EM data collection, refinement and validation statistics for GluN1b-GluN2B NMDA receptor –Fab5 complex

	Active (EMDB-25849) (PDB 7TEQ)	Non-active2 (EMDB-25850) (PDB 7TER)	Non-active1 (EMDB-25851) (PDB 7TES)	Non-active2-like (EMDB-25852) (PDB 7TET)
**Data collection and processing**				
Magnification	105,000	105,000	105,000	105,000
Voltage (kV)	300	300	300	300
Electron exposure (e–/Å^2)	65	65	65	65
Defocus range (μm)	1.5 – 3	1.5 – 3	1.5 – 3	1.5 – 3
Pixel size (Å)	1.37	1.37	1.37	1.37
Symmetry imposed	C2	C2	C2	C2
Initial particle images (no.)	239,873	239,873	239,873	239,873
Final particle images (no.)	13,386	39,661	53,467	74,886
Map resolution (Å)	7.51	5.23	4.70	4.45
FSC threshold	0.143	0.143	0.143	0.143
Map resolution range (Å)				
**Refinement**				
Initial model used (PDB code)	6CNA Crystal structure of Fab5	6CNA Crystal structure of Fab5	6CNA Crystal structure of Fab5	6CNA Crystal structure of Fab5
Model resolution (Å)	8.66	6.92	6.32	5.13
FSC threshold	0.5	0.5	0.5	0.5
Model resolution range (Å)				
Map sharpening *B* factor (Å^2)	-90	-90	-90	-90
Model composition				
Non-hydrogen atoms	27,140	31,560	31,306	28,088
Protein residues	3,442	4,018	3,986	3,563
Ligands	0	0	0	0
CC map vs. model (%)	78	79	77	69
R.m.s. deviations				
Bond lengths (Å)	0.005	0.004	0.004	0.005
Bond angles (°)	0.990	0.926	0.931	1.032
Validation				
MolProbity score	2.10	2.12	2.08	2.08
Clashscore	11.01	11.26	8.98	9.46
Poor rotamers (%)	0.34	1.03	1.27	0.78
Ramachandran plot				
Favored (%)	90.18	90.15	90.89	88.91
Allowed (%)	9.50	9.55	1.27	10.63
Disallowed (%)	0.33	0.30	0.25	0.46

Supplementary Table 4. Primers for cloning and subcloning

Primer Name	Sequence	Company	Purpose
MHC_F_EcoRI	GCGCGCGAATTCTTATGGGGGTGTCGTTTTGGC	Sigma	Heavy chain cloning from hybridoma
MHV_B1_XbaI	GCGCGCTCTAGACGATGTGAAGCTGCAGGAGTC	Sigma	Heavy chain cloning from hybridoma
MHV_B2_XbaI	GCGCGCTCTAGACCAGGTGCAGCTGAAGGAGTC	Sigma	Heavy chain cloning from hybridoma
MHV_B3_XbaI	GCGCGCTCTAGACCAGGTGCAGCTGAAGCAGTC	Sigma	Heavy chain cloning from hybridoma
MHV_B4_XbaI	GCGCGCTCTAGACCAGGTTACTCTGAAAGAGTC	Sigma	Heavy chain cloning from hybridoma
MHV_B5_XbaI	GCGCGCTCTAGACGAGGTCCAGCTGCAACAATCT	Sigma	Heavy chain cloning from hybridoma
MHV_B6_XbaI	GCGCGCTCTAGACGAGGTCCAGCTGCAGCAGTC	Sigma	Heavy chain cloning from hybridoma
MHV_B7_XbaI	GCGCGCTCTAGACCAGGTCCAACTGCAGCAGCCT	Sigma	Heavy chain cloning from hybridoma
MHV_B8_XbaI	GCGCGCTCTAGACGAGGTGAAGCTGGTGGAGTC	Sigma	Heavy chain cloning from hybridoma
MHV_B9_XbaI	GCGCGCTCTAGACGAGGTGAAGCTGGTGGAATC	Sigma	Heavy chain cloning from hybridoma
MHV_B10_XbaI	GCGCGCTCTAGACGATGTGAACTTGGAAGTGTC	Sigma	Heavy chain cloning from hybridoma
MHV_B12_XbaI	GCGCGCTCTAGACGAGGTGCAGCTGGAGGAGTC	Sigma	Heavy chain cloning from hybridoma
MKC_F_EcoRI	GCGCGCGAATTCTTAGGATACAGTTGGTGCAGCATC	Sigma	Light Kappa chain cloning from hybridoma
MKV_B1_XbaI	GCGCGCTCTAGACGATGTTTTGATGACCCAAACT	Sigma	Light Kappa chain cloning from hybridoma
MKV_B2_XbaI	GCGCGCTCTAGACGATATTGTGATGACGCAGGCT	Sigma	Light Kappa chain cloning from hybridoma
MKV_B3_XbaI	GCGCGCTCTAGACGATATTGTGATAACCCAG	Sigma	Light Kappa chain cloning from hybridoma
MKV_B4_XbaI	GCGCGCTCTAGACGACATTGTGCTGACCCAATCT	Sigma	Light Kappa chain cloning from hybridoma
MKV_B5_XbaI	GCGCGCTCTAGACGACATTGTGATGACCCAGTCT	Sigma	Light Kappa chain cloning from hybridoma
MKV_B6_XbaI	GCGCGCTCTAGACGATATTGTGCTAACTCAGTCT	Sigma	Light Kappa chain cloning from hybridoma
MKV_B7_XbaI	GCGCGCTCTAGACGATATCCAGATGACACAGACT	Sigma	Light Kappa chain cloning from hybridoma
MKV_B8_XbaI	GCGCGCTCTAGACGACATCCAGCTGACTCAGTCT	Sigma	Light Kappa chain cloning from hybridoma
MKV_B9_XbaI	GCGCGCTCTAGACCAAATTGTTCTCACCCAGTCT	Sigma	Light Kappa chain cloning from hybridoma
MKV_B10_XbaI	GCGCGCTCTAGACAGACATTCTGATGACCCAGTCT	Sigma	Light Kappa chain cloning from hybridoma
MLC_F_EcoRI	GCGCGCGAATTCTTAGGTGAGTGTGGGAGTGGACTTGG GCTG	Sigma	Light Lambda chain cloning from hybridoma
MLV_B_XbaI	GCGCGCTCTAGACCAGGCTGTTGTGACTCAGGAA	Sigma	Light Lambda chain cloning from hybridoma
pNC-His_FW	CGCTTGCAGGATTCGG	Sigma	Subcloning into pNC-HisT
pNC-His_REV	CAATGTAATTGTTCCCTACTGC	Sigma	Subcloning into pNC-HisT
