# Supplementary material for: Development and characterization of functional antibodies targeting NMDA receptors
Source: Nat Commun. 2022 Feb 17;13:923. doi: 10.1038/s41467-022-28559-3 (PMC8854693; doi:10.1038/s41467-022-28559-3)
Supplement: Supplementary file 3 — Reporting Summary [file 41467_2022_28559_MOESM3_ESM.pdf]

nature research

Corresponding author(s): Hiro Furukawa, Albert Lau

Last updated by author(s): Jan 9, 2022

# Reporting Summary

Nature Research wishes to improve the reproducibility of the work that we publish. This form provides structure for consistency and transparency in reporting. For further information on Nature Research policies, see our Editorial Policies and the Editorial Policy Checklist.

## Statistics

For all statistical analyses, confirm that the following items are present in the figure legend, table legend, main text, or Methods section.

| n/a | Confirmed | |
|---|---|---|
| ☐ | ☒ | The exact sample size (*n*) for each experimental group/condition, given as a discrete number and unit of measurement |
| ☐ | ☒ | A statement on whether measurements were taken from distinct samples or whether the same sample was measured repeatedly |
| ☒ | ☐ | The statistical test(s) used AND whether they are one- or two-sided<br>*Only common tests should be described solely by name; describe more complex techniques in the Methods section.* |
| ☐ | ☒ | A description of all covariates tested |
| ☒ | ☐ | A description of any assumptions or corrections, such as tests of normality and adjustment for multiple comparisons |
| ☐ | ☒ | A full description of the statistical parameters including central tendency (e.g. means) or other basic estimates (e.g. regression coefficient) AND variation (e.g. standard deviation) or associated estimates of uncertainty (e.g. confidence intervals) |
| ☒ | ☐ | For null hypothesis testing, the test statistic (e.g. *F*, *t*, *r*) with confidence intervals, effect sizes, degrees of freedom and *P* value noted<br>*Give P values as exact values whenever suitable.* |
| ☒ | ☐ | For Bayesian analysis, information on the choice of priors and Markov chain Monte Carlo settings |
| ☒ | ☐ | For hierarchical and complex designs, identification of the appropriate level for tests and full reporting of outcomes |
| ☒ | ☐ | Estimates of effect sizes (e.g. Cohen's *d*, Pearson's *r*), indicating how they were calculated |

*Our web collection on statistics for biologists contains articles on many of the points above.*

## Software and code

Policy information about availability of computer code

| | |
|---|---|
| Data collection | Serial EM for cryo-EM data collection. PatchMaster for electrophysiology (TECV) data collection. pClamp10 for patch-clamp electrophysiology. |
| Data analysis | WARP, cisTEM (cryo-EM). Phenix (model refinement). Clampex 11.0 (analysis of patch-clamp electrophysiology) |

For manuscripts utilizing custom algorithms or software that are central to the research but not yet described in published literature, software must be made available to editors and reviewers. We strongly encourage code deposition in a community repository (e.g. GitHub). See the Nature Research guidelines for submitting code & software for further information.

## Data

Policy information about availability of data

All manuscripts must include a data availability statement. This statement should provide the following information, where applicable:

- Accession codes, unique identifiers, or web links for publicly available datasets
- A list of figures that have associated raw data
- A description of any restrictions on data availability

Cryo-EM density maps and atomic coordinates for NMDAR-Fab2, NMDAR-Fab5 have been deposited in the electron microscopy data bank and the Protein Data Bank. For NMDAR-Fab2 the accession codes are EMD-25843 [https://wwwdev.ebi.ac.uk/emdb/EMD-25843]/PDB 7TE9 [https://www.rcsb.org/structure/7TE9], EMD-25844 [https://wwwdev.ebi.ac.uk/emdb/EMD-25844]/PDB 7TEB [https://www.rcsb.org/structure/7TEB], and EMD-25845 [[https://wwwdev.ebi.ac.uk/emdb/EMD-25845] /PDB 7TEE [https://www.rcsb.org/structure/7TEE] for non-active1, non-active1-like, and non-active2-like conformations, respectively. For NMDAR-Fab5 they are EMD-25849 [https://wwwdev.ebi.ac.uk/emdb/EMD-25849]/PDB 7TEQ [https://www.rcsb.org/structure/7TEQ], EMD-25850 [https://wwwdev.ebi.ac.uk/emdb/EMD-25850]/PDB 7TER [https://www.rcsb.org/structure/7TER], EMD-25851 [https://wwwdev.ebi.ac.uk/emdb/EMD-25851]/PDB 7TES [https://www.rcsb.org/structure/7TES], and EMD-25852 [https://wwwdev.ebi.ac.uk/emdb/EMD-25852]/PDB 7TET [https://www.rcsb.org/structure/7TET], for active, non-active2, non-active1, and non-active2-like conformations, respectively. X-ray crystallographic data and coordinates of Fab2 and GluN1b-2B ATD-Fab5

have been deposited to the Protein Data Bank (PDB) under accession codes 7TE4 [https://www.rcsb.org/structure/7TE4] and 7TE6 [https://www.rcsb.org/structure/7TE6], respectively.

# Field-specific reporting

Please select the one below that is the best fit for your research. If you are not sure, read the appropriate sections before making your selection.

☒ Life sciences ☐ Behavioural & social sciences ☐ Ecological, evolutionary & environmental sciences

For a reference copy of the document with all sections, see nature.com/documents/nr-reporting-summary-flat.pdf

# Life sciences study design

All studies must disclose on these points even when the disclosure is negative.

| | |
|---|---|
| Sample size | X-ray diffraction data was collected until radiation damage of crystals weakened the diffraction data to maximize the data quality. We tested more than 50 crystals and conducted structural analyses on the best diffracting crystals which are listed in Supplemental Table 2. For cryo-EM, we collected as many image as possible during the given time slot to maximize the number of particles subjected to single-particle analysis. For electrophysiology, the sample size (n = 4-5) was determined based on the observed data consistency/variability. experimental observation |
| Data exclusions | Electrophysiology recording on Xenopus oocytes/HEK293 cells were excluded when there are artifacts caused byy unhealthy HEK293 cells and Xenopus oocytes, as evidenced by drifting baseline or other problems. |
| Replication | At least 4-5 independent electrophysiology recordings were collected per experiment. These recordings were done on different Xenopus oocytes and HEK293 cells, for TEVC and patch-clamp, respectively. |
| Randomization | Not relevant as no experimental groups were used in this study. |
| Blinding | Blinding is not feasible for cryo-EM and x-ray crystallographic studies. Blinding was applied for electrophysiological data for subjective assessment. |

# Reporting for specific materials, systems and methods

We require information from authors about some types of materials, experimental systems and methods used in many studies. Here, indicate whether each material, system or method listed is relevant to your study. If you are not sure if a list item applies to your research, read the appropriate section before selecting a response.

## Materials & experimental systems

| n/a | Involved in the study | |
|---|---|---|
| ☐ | ☒ | Antibodies |
| ☐ | ☒ | Eukaryotic cell lines |
| ☒ | ☐ | Palaeontology and archaeology |
| ☒ | ☐ | Animals and other organisms |
| ☒ | ☐ | Human research participants |
| ☒ | ☐ | Clinical data |
| ☒ | ☐ | Dual use research of concern |

## Methods

| n/a | Involved in the study | |
|---|---|---|
| ☒ | ☐ | ChIP-seq |
| ☒ | ☐ | Flow cytometry |
| ☒ | ☐ | MRI-based neuroimaging |

## Antibodies

| | |
|---|---|
| Antibodies used | The antibodies in this study were made in-house by mouse immunization with the GluN1a-2B NMDAR proteins. |
| Validation | The binding of antibodies were validated by single-particle cryo-EM. |

## Eukaryotic cell lines

Policy information about cell lines

| | |
|---|---|
| Cell line source(s) | HEK293 (ATDD, HEK293T (ATCC), Sf9 (Thermo Fisher). |
| Authentication | The cell lines above are not authenticated. |
| Mycoplasma contamination | All cell lines were confirmed to be negative for mycoplasma. |

| Commonly misidentified lines (See ICLAC register) | No commonly misidentified cell lines were used. |
| --- | --- |
